# Morphological study on the effects of sample size on ovarian tissue vitrification

**DOI:** 10.5935/1518-0557.20210075

**Published:** 2022

**Authors:** Ágata Dupont, Eduardo Sanguinet, Maitê Ferreira, Larissa Ramos, Nivia Lothhammer, Nilo Frantz, Adriana Bos-Mikich

**Affiliations:** 1 Department of Morphological Sciences, ICBS, Federal University of Rio Grande do Sul, Porto Alegre, RS, Brazil; 2 Nilo Frantz Reproductive Medicine, Porto Alegre, RS, Brazil

**Keywords:** ovarian cortex, vitrification, sample size, follicle, stroma, morphology

## Abstract

**Objective:**

The present study aimed to assess the effects of ovarian cortex sample size on tissue morphological integrity after vitrification in a metal capsule.

**Methods:**

Bovine ovarian tissue samples cut in large and small fragments (1x1x5 and 1x1x3 mm, respectively - 5 and 3 mm refer to length), vitrified in a metal capsule were fixed for histological analysis immediately after rewarming or after 48 hours culture. We assessed primordial, primary and secondary follicle morphology and stromal integrity.

**Results:**

Primordial follicles showed the highest rates of normal morphology after rewarming and after 48 hours culture in both, small and large tissue fragments. Primary follicles presented a significant drop in normal morphology in large samples, after 48 hours in culture. Stromal integrity was well-preserved immediately after rewarming in small and large fragments but presented a significant drop in normal morphology in large samples, after 48 hours in culture.

**Conclusions:**

The ovarian reserve, represented by Primordial follicles, is well-preserved in small or large fragments, after vitrification and culture. However, the stromal components present better preservation after vitrification\rewarming, when tissue samples are cut in small fragments. Thus, small cortex samples should be preferred for ovarian tissue vitrification.

## INTRODUCTION

Cryopreservation of ovarian tissue is an important alternative for fertility preservation of young women and girls affected by an oncological condition. Two methods are commonly used to cryopreserve and store biological material, namely, slow freezing and vitrification. One major problem for researchers working on female fertility preservation is the fact that many years may pass, before the patients require an auto-transplantation, and the outcome of the cryopreservation technique is reported. Thus, it is difficult for the clinicians to decide which cryopreservation method to use based on tissue survival after re-transplantation. In this scenario, several authors have chosen histological and/or biochemical evaluations to assess tissue integrity post-thawing or post-rewarming (Candy & Wood, 1997; Fabbri *et al*., 2003; Wang *et al*., 2008; Keros *et al*., 2009).

Different factors involved in successful cryopreservation of ovarian tissue, such as cryoprotectants, rapid or slow cooling and vitrification have been extensively investigated. However, few studies have paid attention to the size of the fragment, particularly for the vitrification procedure. It is almost a consensus that small, 1 x 1 x 2mm (length, width, height, respectively) fragments should be better for cryoprotectant diffusion in and out of the tissue. An early study by our group (Ferreira *et al*., 2010) showed that follicular survival was higher, when the frozen\thawed tissue sample is cut in small fragments, presenting at least one of its dimensions ≤2mm. On the other hand, larger tissue samples would be easier to cut and to handle during the preparation process and the follicular gain would be higher (Jeremias *et al*., 2003). Early experiments by Ferreira *et al*. (2010), were performed using the slow freezing procedure and, to our knowledge, there is no similar research on the impacts of tissue sample size on the follicular and stroma integrity after vitrification/rewarming technique. However, it has been shown that the antioxidant defense capacity and tissue viability have similar values for vitrified ovarian tissue cut in small or large fragments, immediately after rewarming and after a 48h culture (Massignam *et al*., 2018).

In addition, research on ovarian tissue morphology after cryopreservation shouldn´t focus merely on the follicular population of the biological sample. It is acknowledged, that the stromal components of the ovarian cortex play important roles on the survival and maintenance of the follicular pool viability after tissue grafting upon re-transplantation (Sheikhi *et al*., 2011; Rodrigues *et al*., 2013). Thus, in addition to follicular integrity, an important factor to be taken into consideration, when performing ovarian tissue cryopreservation, is to preserve the integrity of the stromal components found in the tissue sample.

The present study aimed to compare the morphology of follicles and stroma in gonadal fragments cut into two-fragment sizes, after vitrification/rewarming and *in vitro* 48h-culture.

## MATERIAL AND METHODS

### Tissue processing and vitrification

Bovine ovaries were collected at a local abattoir and transported to the laboratory within two hours of slaughter, in a sterile vessel containing saline solution at room temperature (RT; ~23ºC). The Institutional Ethics Committee of the Federal University of Rio Grande do Sul approved the study (Permit no. 25088).

The vitrification protocol followed the procedures described earlier (Aquino *et al*., 2014). The ovarian cortex was cut in several thin stripes (1 mm high x 1 mm width), and then in two different lengths: group 1 samples were 1x1x3 mm (small fragments, SF) and group 2 samples were 1x1x5 mm (large fragments, LF). Three- and 5-mm measures refer to the length of the fragment. The specimens were transferred first to an equilibrium solution (ES) with 7.5% ethylene glycol (EG) and DMSO, and then to a vitrification solution (VS) with 15% EG and DMSO, both in HTF (Irvine) medium for 25 and 15 minutes (min), respectively, at room temperature. Ten to 12 SF and LF fragments, from different ovaries, were placed in the bottom of a metal cryovial (Patent no.: BR 20 2013 019739 0) (Bos-Mikich *et al*., 2012; Aquino *et al*., 2014); a lid was tightly fastened on the top of the vial and the system was immersed in liquid nitrogen (LN2). For each experimental replicate, four to five fresh samples from different ovaries were fixed with 4% paraformaldehyde (PFA) for 15 min., before processing for histology. The experiments were performed in triplicates.

### Tissue rewarming and culture

The metal cryovials were removed from the LN2 and exposed to tap water, at room temperature, for 30 seconds to allow the lids to be unfastened. The bottom of the cryovial was immersed in a water bath at 37ºC for 1 minute to rewarm the contents. The rewarmed tissue fragments were gently transferred to the first warming solution (S1) containing 1M sucrose for 1 min, followed by the second solution (S2) containing 0.5M sucrose for 3 min, and the last solution contained 0.25M sucrose for 5 minutes. Solutions were at room temperature. After rewarming, the fragments were cultured for 48 hours in an HTF medium supplemented with 20% synthetic serum substitute (SSS; Irvine Scientific) at 38.5ºC. Fresh tissue samples were placed under the same culture conditions. After the 48-hour culture, the ovarian tissue fragments from the different groups were fixed with 4% PFA and processed according to standard histology protocols. The tissue sections were stained with Hematoxylin and Eosin (HE).

The follicles were classified according to their stage of development in: Primordial - surrounded by a single layer of flattened granulosa cells; Primary - surrounded by one layer of cuboidal granulosa cells, Secondary - surrounded by more than one layer of cells with no antrum (Gougeon, 1996). Criteria for considering damaged primordial or primary follicles included: presence of a vacuole in the ooplasm, pyknotic germinal vesicles, retracted ooplasm and clear spaces between follicular cells and follicle basement membrane. Only primordial and primary follicles were considered for statistical analysis, because they are directly related to the ovarian reserve. We present data on the morphology of secondary follicles as they are part of theoverall tissue integrity.

A qualitative evaluation of the stromal morphology was performed after a 48-hour culture. Stroma damage was classified according to the proportion (%) of altered tissue, seen at with 10X low power magnification in the field of view, for each tissue slice (histological cuts) in 0 to 10% alterations (small area of alterations), 20 to 80% (medium area of alterations) and 90 to 100% (completely altered tissue slice). Tissue normality was assessed according to the shape and dispersion of the collagen fibers: thread-like appearance, with uniform distribution and no gaps among them; normal, fibroblasts and interstitial-cells nuclei (no pyknotic, shrunken nuclei). All samples were analyzed in a blind format.

### Statistical analyses

Follicular morphological integrity was analyzed by analysis of variance (ANOVA), performed using the *Minitab*^®^ Statistical Software for Windows (version 19.2). We used the chi-square to compare values of follicle and stromal integrity. Differences with *p*<0.05 were considered statistically significant.

## RESULTS

### Follicular quality

Table 1 shows data on the 1043 follicular structures analyzed and classified into normal or damaged primordial, primary and secondary follicles. Regarding primordial follicles normal morphology, there was no statistical difference among the vitrified groups, vitrified cultured for 48 hours and fresh samples cultured for 48 hours. Fresh small fragments presented the highest percentage of damaged primordial follicles among all groups. Vitrified small samples preserved equally well primordial and primary follicles. After 48 hours of culture, there was a significant drop in the rate of primary follicle normal morphology when compared to primordial follicles, but the percentage was still very high. Large tissue samples showed significant differences in normal primordial and primary follicle morphology, both immediately after vitrification and after a 48-hour culture. Primary follicles presented the lowest rate of normal morphology in vitrified large fragments, cultured for 48 hours. There was no statistical difference among the other groups. Despite the small number of secondary follicles present in the fragments, one could see a decrease in normal morphology in all experimental groups, when compared with fresh controls. In the vitrified large sample group, there was a significantly higher normal morphology rate of primordial follicles, when compared with primary structures. After 48 hours in culture, both small and large vitrified fragments presented a higher rate of normal morphology primordial follicles, when compared with primary follicles. Fresh cultured small and large fragments presented similar morphological integrity rates between the primordial and primary follicles. Considering the four cryopreserved groups, the proportion of morphologically normal primordial and primary follicles was very high, except for the group of large samples cultured for 48 hours after rewarming. In this group, primary follicles were negatively affected by the culture period in the vitrified large fragment group. It is interesting to point out that after 48 hours in culture, vitrified small and large fragments presented a similar behavior for the proportions of morphologically intact primordial, primary and secondary follicles, being that the primordial type was the best preserved in both sample size groups.

**Table 1.** Numbers (n) and percentages (%) of morphologically normal fresh and vitrified primordial, primary and secondary follicles, with and without 48h culture.

Tissue sample	Normal primordial n/Total (%)	Normal primary n/Total (%)	Normal secondary n/Total (%)
**Fr SF**	58/87 (66.7)^aA^	113/138 (81.9)^aB^	21/26 (95.5)^aC^
**Fr LF**	72/76 (94.7)^bA^	237/244 (97.1)^bA^	29/31 (93.5)^aA^
**Vitr SF**	5/5 (100)^cA^	29/29 (100)^bA^	5/6 (83.3)^bB^
**Vitr LF**	34/34 (100)^cA^	114/121 (94.2)^bB^	7/17 (41.2)^cC^
**Fr SF 48h**	10/10 (100)^cA^	69/70 (98.6)^bA^	1/1 (100)^dA^
**Fr LF 48h**	59/61(96.7)^cA^	99/103 (96.1)^bA^	7/10 (70)^eB^
**Vitr SF 48h**	31/31 (100)^cA^	127/134 (94.8)^bB^	12/23 (52.2)^cC^
**Vitr LF 48h**	2/2 (100)^cA^	8/13 (61.5)^cB^	6/8 (75)^bC^

Fr SF=fresh small fragment, Fr LF=fresh large fragment, Vitr SF=vitrified small fragment, Vitr LF=vitrified large fragment, Fr SF 48h=fresh small fragment 48h culture, Fr LF 48h=fresh large fragment 48h culture, Vitr SF 48h=Vitrified small 48h culture, Vit. LF 48h=Vitrified large 48h culture. n=absolute number/ Total number (%, percentage) Different low case letters indicate significant differences in the same column; different high case letters indicate significant differences in the same line.

### Stromal integrity

**Figure 1:**
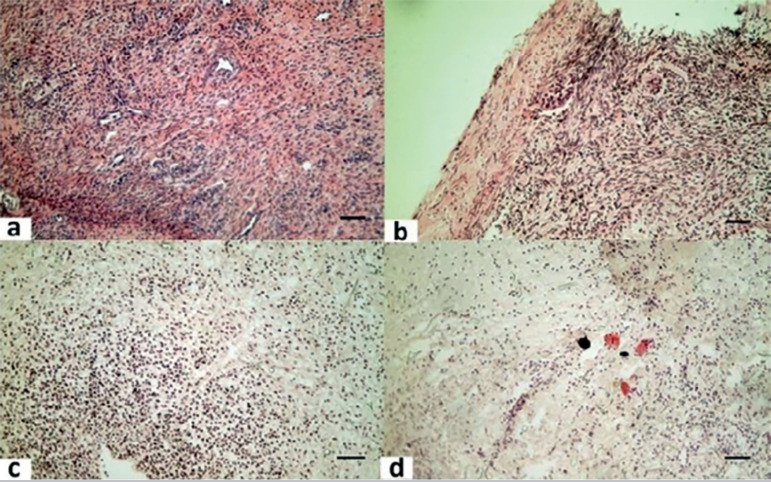
Illustrative images of fresh and vitrified\rewarmed ovarian tissue slices stained with Hematoxylin and Eosin (HE). (a) Fresh and (b) rewarmed intact stromal structure, (c) 20-80% and (d) 90-100% damaged stroma. 10X, bar= 10µ*m*.

The amount of stromal damage was qualitatively analyzed on a total of 254 tissue slices from fresh and vitrified\rewarmed ovarian fragments after 48 hours in culture (Figure 1). Table 2 shows that there was a deleterious effect of the cryopreservation process on stromal components of small and large fragments, when compared with fresh controls. However, there was a significantly higher proportion of damage on large samples after cryopreservation, when compared with the damage seen in vitrified small tissue samples.

**Table 2.** Stromal damage in fresh and rewarmed ovarian tissue samples after 48hrs culture.

% Stromal damage	Nº Vit SF 48h (%)	Nº Fr LF 48h (%)	Nº Vit LF 48h (%)	Nº Fr SF 48h (%)
0-10	28 (85)^a^	29 (86)^a^	57 (77)^a^	22 (19)^b^
20-80	5 (15)^a^	5 (14)^a^	7 (9)^a^	30 (27)^b^
90-100	0 (0)^a^	0 (0)^a^	10 (14)^b^	61 (54)^c^

Nº Fr SF 48h=Number of samples fresh small fragments 48 h culture, Nº Fr LF 48h=Number of samples fresh large fragments 48 h culture, Nº Vit SF 48h=Number of samples vitrified small fragments 48 h culture, Nº Vit LF 48h=Number of samples vitrified large fragments 48 h culture. Different low case letters indicate significant differences in the same line.

## DISCUSSION

The present study investigated the effects of sample size on ovarian tissue integrity after vitrification\rewarming and 48 hours in culture. After rewarming, the results showed an overall better normal morphology of the primordial, compared with the primary follicles, an outcome similar to that of previous reports (Candy & Wood, 1997; Chen et al., 2006; Aquino et al., 2014). These observations suggest that the primary follicle structure composed of cuboidal cells, in contrast to the flattened cells of the primordial follicle, may be more sensitive to the cryopreservation process, as suggested by Chen et al. (2006), after slow freezing of human ovarian tissue. In addition, the size of the fragment did not affect the primordial follicles' normal morphology, neither immediately after rewarming, nor after 48h in culture, indicating that the earliest follicle stage recovers well from cryopreservation, independent of sample size. On the other hand, stromal components were affected by sample size, where the larger fragments presented the lowest normal morphology rates after rewarming. Ferreira et al. (2010) showed that there is a significant increase in the relative risk of abnormal follicle morphology after slow freezing, when the tissue fragment is cut with all dimensions larger than 2 mm. However, the authors did not distinguish between primordial and primary follicle population, showing an overall decrease in follicular normal morphology in large tissue samples.

One logical reason to cut ovarian samples in small fragments is the fact that to achieve an effective cryoprotection, the cryoprotective agent should diffuse to the innermost cells and extracellular components of the ovarian tissue. Smaller samples may benefit from an adequate, short equilibration time as a prolonged exposure to cryoprotectant agents, necessary to perfuse into a large fragment, may be cytotoxic. Thus, as stated by Yavin & Arav (2007), sample volume is an essential factor for successful vitrification, the smaller the tissue sample, the less liquid is necessary to cool it and the lower is the probability of ice-crystal formation and tissue damage. A recent report described the use of a dissecting tool for slicing ovarian tissue in fragments measuring 1 x 7 x 7 mm, prior to cryopreservation (Almodin *et al*., 2020). After vitrification using the Vitroequip procedure, the authors described no statistical differences in follicular morphology between fresh and cryopreserved tissue samples, showing that relatively large fragments shall be used for cryostorage of human ovarian cortex samples.

There was a very low follicular count in some experimental groups, such as the vitrified large 48-hour culture or the vitrified small fragment groups. In a careful analysis of human ovarian cortex samples taken from 17 patients, Schmidt *et al*. (2003) found that the follicular population of ovarian cortex samples were unevenly distributed and there was a huge variation in follicular density among tissue fragments. The authors described that in a group of 15 samples from one patient, with similar tissue volumes, one fragment contained two follicles and the other 2,497. Although our study was performed using bovine ovarian samples, we believe that the observation described in their study represents a plausible explanation for the low numbers of follicular structures in some of our experimental groups, even though each group contains tissue samples from three experimental repeats.

Stromal integrity represents a key factor for follicular survival at re-transplantation. Our results agree with those from previous reports (Keros *et al*., 2009; Sheikhi *et al*., 2011; Bos-Mikich *et al.,* 2012; Aquino *et al*., 2014), showing that vitrification preserves well the fibrous and cellular components of the ovarian stroma. There was, however, a drop on stromal integrity after the 48-hour culture of the large tissue fragments. This was an unexpected finding considering that previous studies measuring the rate of tissue necrosis by lactate dehydrogenase levels, did not detect any difference between tissue size and between vitrified and fresh samples after 48 hours in culture (Massignam *et al*., 2018). One explanation for the decrease in tissue integrity in large samples in the present study may be that the histology technique used to analyze the samples should be adjusted for larger tissue fragments exposed to a cryopreservation process. Example of that would be the fixative period, in which a standard time was used for all experimental groups in the present study. Inadequate fixation time, not adjusted to different sample sizes, may have caused an inadequate paraffin embedding which causes impaired tissue morphology.

Slow freezing is the preferred cryopreservation method in fertility preservation programs around the world. At present, cryopreservation of ovarian tissue has made possible the birth of more than 130 babies, of which only two were reported after vitrification of ovarian cortex fragments (Kawamura *et al*., 2013; Suzuki *et al*., 2015; Rivas Leonel *et al*., 2019). We believe that results presented in this study and former reports using vitrification (Keros *et al*., 2009; Sheikhi *et al*., 2011; Wang *et al*., 2008; Amorim *et al*., 2011; Corral *et al*., 2018) for the preservation of ovarian tissue will encourage physicians and embryologists to consider the use vitrification for fertility preservation. Vitrification has basically replaced slow freezing for human embryo and oocyte cryopreservation worldwide. This is primarily due to its success rates after rewarming in terms of fertilization, embryo development, gestation and birth. It is simple to perform and bear low costs.

The metal capsule employed in this study enables very fast cooling rates, an essential factor for efficient vitrification, and the silicone ring placed in the lid of the capsule enables a perfect seal of the system to be used in clinical grade fertility preservation programs. The capsule is simple to use and has shown very good preservation of both, follicular and stromal components of the cryopreserved tissues, at rewarming and after 48 hours in culture (Bos-Mikich *et al*., 2012; Aquino *et al*., 2014).

The goal of a fertility preservation program should focus on the maintenance of a viable ovarian reserve, represented by Primordial follicles, and the surrounding stroma. The results here presented using the bovine ovarian tissue model showed that Primordial follicles and stroma are well-preserved after vitrification in the metal capsule and 48 hours culture, being that smaller fragments of the ovarian cortex should be preferred for tissue banking. Similar studies are being performed on human ovarian cortex samples to investigate the feasibility of the methodology for female fertility preservation programs.
